# Machine Learning Models for Predicting Mental Health Crises in Adolescents Using Electronic Health Records: A Systematic Review

**DOI:** 10.7759/cureus.89873

**Published:** 2025-08-12

**Authors:** Abdulkreem Al-Juhani, Rodan Desoky, Ziyad Iskander, Rimaz M Alotaibi, Nouf N Alzain, Naif Aljohani, Mamoun M Alrefaai, Raneen A Alhasanat, Manar A AlMehaimeed, Abdulrahman S Alharthi

**Affiliations:** 1 Forensic Medicine, Forensic Medicine Center, Jeddah, SAU; 2 Medicine and Surgery, College of Medicine Alfaisal University, Riyadh, SAU; 3 Preventive Medicine, Taibah University, Madinah, SAU; 4 Forensic Medicine, Almaarefa University, Diriyah, SAU; 5 Family Medicine, Almaarefa University, Diriyah, SAU; 6 Internal Medicine, Almaarefa University, Diriyah, SAU; 7 Medicine and Surgery, Almaarefa University, Diriyah, SAU; 8 Medicine and Surgery, College of Medicine, Qassim University, Buraydah, SAU; 9 Medicine and Surgery, College of Medicine, Umm Al-Qura University, Makkah, SAU

**Keywords:** adolescent mental health, electronic health records, machine learning, natural language processing, psychiatric hospitalization, risk stratification, self-harm detection, suicide prediction

## Abstract

The incidence of suicide, self-harm, and mental crises among teenagers is rising, presenting significant global public health issues. Conventional clinical risk evaluations have inadequate predictive accuracy, often overlooking high-risk adolescents. Machine learning models employing electronic health records provide an innovative method for predicting mental health crises through the integration of intricate clinical data patterns. Hence, this review aims to comprehensively examine and synthesize information about machine learning models created using electronic health record data for predicting suicide attempts, self-harm, or mental hospitalization in teenagers. Adhering to Preferred Reporting Items for Systematic reviews and Meta-Analyses (PRISMA) principles, we executed an exhaustive search across six databases (2000-2025) for peer-reviewed research utilizing machine learning algorithms on electronic health record data to predict adolescent mental health crises. The inclusion criteria emphasized structured or unstructured electronic health record inputs, teenage cohorts (ages 10-20), and performance indicators like area under the curve (AUC), sensitivity, or specificity. The Prediction Model Risk of Bias Assessment Tool (PROBAST) was utilized to evaluate the risk of bias. Our search yielded five studies (2019-2024) that satisfied the inclusion criteria. All studies were retrospective cohorts conducted in high-income nations. Structured electronic health record data (e.g., diagnoses, prescriptions) were frequently utilized; two studies included natural language processing. Machine learning models demonstrated moderate to high discrimination (AUC 0.68-0.88), exhibiting optimal performance in short-term suicide prediction with hybrid data inputs. All investigations, however, exhibited a significant risk of bias in the analysis domain owing to insufficient external validation and absent calibration data. The positive predictive values consistently remained modest across all models. Overall, machine learning models demonstrate potential for enhancing adolescent suicide risk classification utilizing electronic health record data, surpassing numerous traditional instruments. Nonetheless, issues of generalizability, ethical constraints, and implementation obstacles remain. Thorough validation, calibration, and equity assessments are necessary prior to incorporation into clinical practice.

## Introduction and background

The escalating prevalence of adolescent mental health crises, encompassing suicide attempts, self-harm, and psychiatric hospitalization, is a significant global issue. Suicide is the second leading cause of mortality among those aged 10 to 19 years. Conventional risk assessments, such as clinician evaluation and static checklists, often fail to precisely identify those at high risk [1].

These instruments generally depend on restricted historical data and have inadequate predictive efficacy, with stated area under the curve (AUC) values frequently below 0.70 [2,3]. Su et al. [1] illustrated that dependence exclusively on previous self-harm history resulted in inadequate predictive efficacy, whereas a machine learning (ML) model incorporating various EHR variables attained markedly enhanced discriminating (AUC 0.74 for self-harm; 0.72 for suicide attempts). ML provides a data-driven approach by utilizing extensive electronic health records (EHRs) to identify intricate risk patterns. In contrast to traditional models, ML algorithms may integrate structured and unstructured clinical data, such as diagnoses, medication histories, encounter patterns, and clinician notes, to provide personalized crisis risk scores [4,5].

Numerous studies have indicated that ML models surpass baseline statistical methods in this field. Garriga et al. [6] created a continuous EHR-based ML model that forecasted mental health crises within 28 days, with an AUC of 0.797 and a sensitivity of 58% at 85% specificity. In pediatric psychiatric populations, ML models utilizing EHR data have attained sensitivities over 80% in detecting kids at risk for post-discharge suicide-related hospitalization [7].

Notwithstanding these improvements, to our knowledge, no previous systematic study has explicitly concentrated on the utilization of ML in EHR data for forecasting adolescent mental health crises. Current reviews have either investigated ML for suicide risk across various age demographics [8] or analyzed ML for general psychiatric outcomes like depression [9], yet none have focused on the convergence of adolescents, EHR-based inputs, ML techniques, and acute crisis outcomes such as suicide attempts or psychiatric hospitalization. 

This represents a severe deficiency, as adolescents markedly differ from adults in developmental pathways, symptomatology, and EHR reporting practices. Consequently, the relevance and efficacy of ML models developed on adult or mixed-age datasets may not directly apply to adolescent care.

This systematic review seeks to address that deficiency by assessing and consolidating the information about ML models utilized on EHR data for forecasting mental health crises in adolescents. This evaluation will concentrate solely on prediction performance and clinical risk assessment to guide the prospective incorporation of ML-based tools into adolescent mental health care.

Methodology

Search Strategy

We adhered to the Preferred Reporting Items for Systematic Reviews and Meta-Analyses (PRISMA) standards in the design and reporting of our review. A thorough search was performed in PubMed, Embase, Scopus, Web of Science, IEEE Xplore, and PsycINFO for research published in English from 2000 to 2025. Search terms integrated synonyms and controlled vocabulary for adolescents (e.g., "adolescent," "youth"), EHRs (e.g., "electronic health record," "EHR"), ML (e.g., "machine learning," "algorithm," "neural network," "random forest," etc.), and the desired outcomes (e.g., "suicide," "self-harm," "psych* hospitali*," etc.). Boolean operators (AND, OR) were employed to connect concepts. For instance, we integrated suicide/self-harm terminology with ML and EHR terminology (e.g., ("suicide" OR "self-harm") AND ("electronic health record" OR "EHR") AND ("machine learning" OR "algorithm") AND ("adolescen*")). Searches were augmented by manually reviewing the reference lists of listed studies.

Inclusion and Exclusion Criteria

Inclusion criteria: Primary, peer-reviewed publications published between 2000 and 2025 in English that documented the construction and/or validation of machine-learning predictive models utilizing EHR data from teenage patients aged about 10 to 20 years. Eligible studies were required to utilize EHR-derived inputs, implement at least one ML algorithm, and forecast outcomes associated with suicide, self-harm (including suicidal ideation or attempts), or mental hospitalization. Only studies that published model performance metrics, such as discrimination or calibration measurements including AUC, accuracy, and sensitivity, were included.

Exclusion criteria: We excluded reviews, systematic reviews, meta-analyses, editorials, commentaries, conference abstracts, preprints, and other non-peer-reviewed materials. Research not concentrated on the 10-20 age demographic (or lacking stratified adolescent data) was omitted. Studies that did not utilize EHR data (e.g., those relying exclusively on questionnaires or imaging), as well as studies without ML model building or reporting of prediction performance, were excluded.

Study Selection

**Figure 1:**
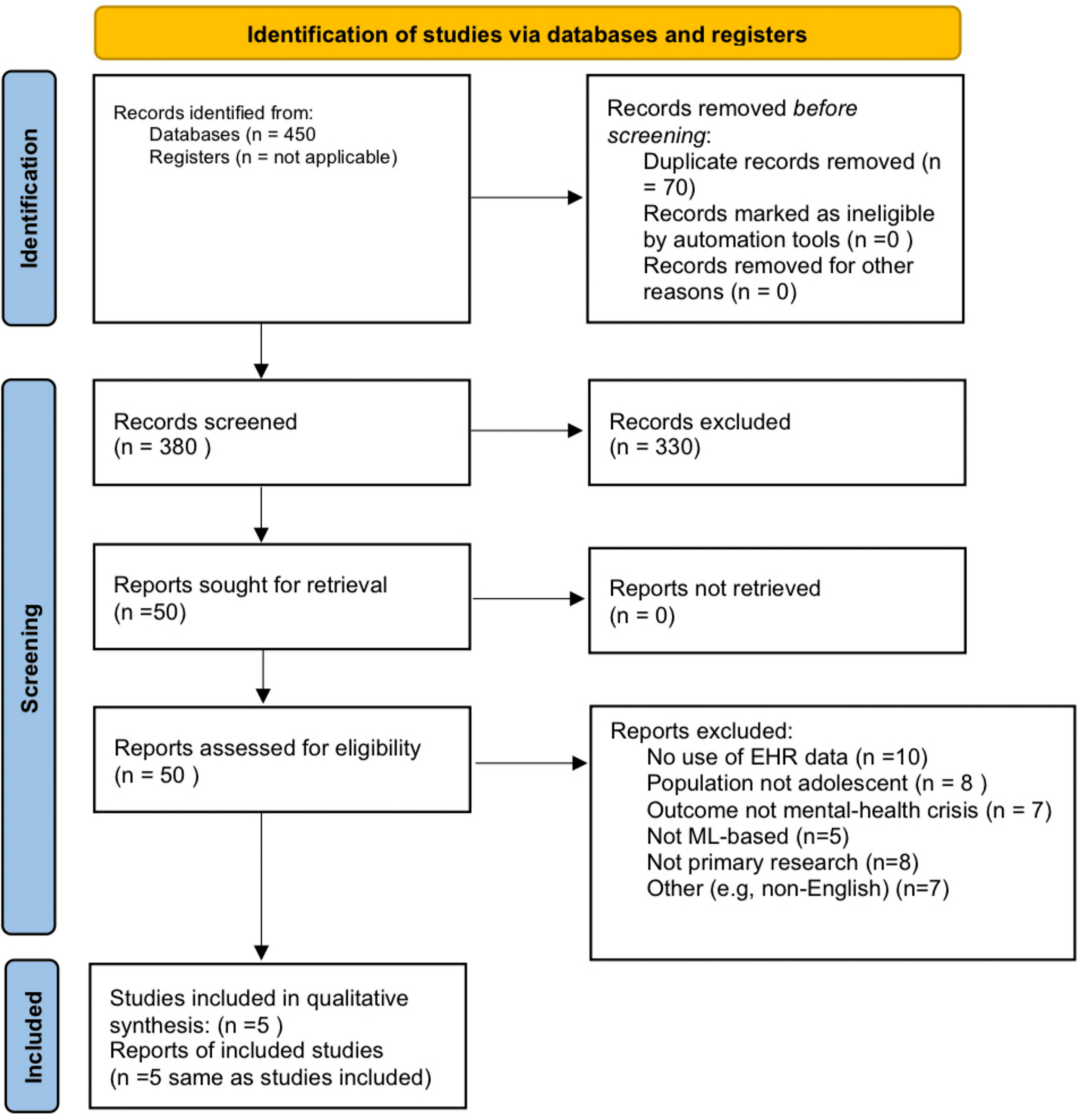
Preferred Reporting Items for Systematic Reviews and Meta-Analyses (PRISMA) flow chart

Two reviewers separately evaluated the titles and abstracts of all retrieved records for potential relevance. Articles that successfully passed title and abstract screening were subjected to full-text examination by the same two reviewers. Disputes over inclusion were settled by conversation and, if required, by engaging a third reviewer. The research selection method adhered to PRISMA 2020 principles. The PRISMA flow diagram (Figure 1) depicting identified, screened, excluded, and eventually included records will be presented. Only papers that fulfilled all inclusion criteria were retained for data extraction.

Data Extraction

Two reviewers separately extracted data utilizing a standardized form. The extracted information encompassed: study identifiers (author, year), study design and setting, population characteristics (age range of adolescents, sample size), specifics of the EHR data source, target outcome (type of suicidal behavior, self-harm, or psychiatric admission), utilized predictors, ML methods (algorithms, feature selection, cross-validation), and reported performance metrics (e.g., AUC, accuracy, sensitivity, specificity, precision, recall). We additionally removed all validation methodologies (internal/external) and pertinent implementation specifics. The data extraction template was guided by existing frameworks, such as the Transparent Reporting of a multivariable prediction model for Individual Prognosis Or Diagnosis (TRIPOD) declaration and the Checklist for critical Appraisal and data extraction for systematic Reviews of prediction Modelling Studies (CHARMS) checklist, to guarantee comprehensiveness. Discrepancies in the extracted data were reconciled through consensus, with a third reviewer consulted as necessary.

Risk of Bias Assessment

The risk of bias in each included study was independently assessed by two reviewers utilizing the Prediction Model Risk of Bias Assessment Tool (PROBAST) technique [10]. PROBAST is explicitly formulated for prediction-model research, encompassing participant domains, predictors, outcomes, and analytical methods. Each study was evaluated based on signaling questions and domain criteria, resulting in assessments of "low," "high," or "unclear" likelihood of bias. Concerns regarding applicability (generalizability to our review question) were also evaluated. Discrepancies in PROBAST ratings were reconciled via conversation.

Synthesis Approach

Due to anticipated variability in study designs, data, and ML methodologies, we refrained from doing a quantitative meta-analysis. We performed a qualitative (narrative) synthesis instead. We compiled essential study attributes, model specifications, and performance outcomes. The findings were categorized by result type (suicide attempt, self-harm, psychiatric admission) and by ML method. We analyzed reported metrics and identified common drivers across trials. We identified tendencies, such as the most effective algorithms, and deficiencies in the literature where applicable. Results are presented descriptively in tables and text to furnish a full overview of ML-based EHR predictions for teenage mental health crises.

Results

A total of 450 records were identified using database searches, and following the elimination of duplicates and abstract screening, 50 full-text articles were evaluated. In all, five papers (published between 2019 and 2025) satisfied all inclusion criteria and were incorporated into the review (PRISMA flow diagram). This seems to be the inaugural systematic evaluation concentrating exclusively on ML models utilizing EHR data for youth suicide, self-harm, and associated crisis outcomes. All studies examined were conducted in high-income nations (four in the United States and one in Australia) and utilized retrospective EHR data to forecast mental health crises in adolescents. The target population in the research consisted of adolescents (ages 10-20, with one study involving children as young as five), and sample sizes varied from approximately 2,700 to over 256,000 persons. The key attributes of these investigations, encompassing context (outpatient versus inpatient), outcome definitions, and ML methodologies, are encapsulated in Table 1. Overall, suicide attempts were the predominant outcome; however, one study investigated suicide-related hospital readmissions, while another concentrated on identifying histories of suicidal behavior (a surrogate for risk) from clinical documentation. All research employed an observational design, specifically retrospective cohort or case-control methodologies utilizing EHR data.

**Table 1:** Study characteristics and ML overview - presents country, setting, sample size, outcome, ML method, and validation CV, cross-validation; EHR, electronic health record; LASSO, Least Absolute Shrinkage and Selection Operator; ML, machine learning; NLP, natural language processing

Study (Author, Year)	Country	Setting	Sample Size (N)	Age Range	Outcome Predicted	EHR Data Type	ML Model(s)	Validation Type	Comparator
Su et al. (2020) [1]	USA	Outpatient	41,721	10-18 years	Suicide attempt	Structured	Logistic regression (L1)	10-fold CV	None
Penfold et al. (2021) [2]	USA	Outpatient	256,823 visits	≤18 years	Suicide attempt (within 90 days)	Structured	LASSO regression	Temporal split	Existing clinical tools
Carson et al. (2024) [3]	USA	Inpatient	2,789	12-20 years	Suicide-related hospitalization (post-discharge)	Structured + unstructured	Gradient boosting, random forest	Train/test split	Random forest baseline
Carson et al. (2019) [4]	USA	Inpatient	73	10-18 years	History of suicide attempt	Unstructured (NLP)	Random forest (NLP)	10-fold CV	None
Tennakoon et al. (2023) [5]	Australia	Outpatient	4,610	5-19 years	Self-harm or suicidal ideation	Structured	LASSO logistic regression	Five-fold CV	None

EHR Data and Feature Engineering

Table 2 illustrates that three of the five models utilized solely structured EHR data, including diagnosis codes, medication records, prior visit history, and demographic information, whereas two studies integrated unstructured text from clinical notes through natural language processing (NLP). For instance, Su et al. [1] utilized diagnoses, pharmaceutical prescriptions, laboratory findings, and visit frequency from the year preceding the prediction point, whereas Penfold et al. [2] incorporated demographics, psychiatric diagnoses, and recent mental health visit data. Tennakoon et al. [5] also utilized structured indicators, including documented diagnoses, previous self-harm incidents, and treatment information. Conversely, Carson et al. [4] analyzed unstructured admission notes to identify records of previous suicide attempts, utilizing text vectorization (bag-of-words) to convert notes into features. Carson et al. [3] integrated both data types, extracting essential phrases from inpatient clinical notes in conjunction with coded information such as diagnoses, laboratory results, and medication history. Feature engineering techniques varied from automated selection approaches to specialized text processing for certain domains. Three studies utilized regularized logistic regression methods that conduct intrinsic feature selection (L1-penalized/Least Absolute Shrinkage and Selection Operator (LASSO) regression). Su et al. [1] expressly employed a sequential forward feature selection method alongside L1 regularization. A vocabulary of salient words was extracted from notes (following the elimination of common stopwords) and utilized as predictors for the NLP-based model. The temporal windows for predictor data differed across studies: the majority of structured-data models employed a look-back period (e.g., 12 months preceding the index date or 90 days following an index visit/discharge) to determine the inclusion of EHR records, while the NLP model examined text from the index hospitalization itself.

**Table 2:** EHR features and data details - structured/unstructured data, feature engineering, and time windows EHR, electronic health record; LASSO, Least Absolute Shrinkage and Selection Operator; NLP, natural language processing

Study	Structured Data Features	Unstructured Data (NLP)	Feature Engineering	Time Window Used
Su et al. (2020) [1]	Diagnoses, medications, labs, visit history	No	Forward selection, L1 regularization	365 days pre-index
Penfold et al. (2021) [2]	Demographics, diagnoses, prior MH visits	No	Penalized regression with time windows	90 days post-visit
Carson et al. (2024) [3]	Diagnoses, labs, med history, structured notes	Yes (clinical notes)	Token extraction, feature aggregation	90 days post-discharge
Carson et al. (2019) [4]	None	Yes (admission notes)	Text vectorization (bag-of-words)	During index hospitalization
Tennakoon et al. (2023) [5]	Diagnoses, previous self-harm, treatment details	No	LASSO-based predictor selection	Up to 12 months follow-up

The study examined various ML algorithms (refer to Table 1). Basic linear models were prevalent; penalized logistic regression with L1 regularization, sometimes termed LASSO models, was employed in three investigations. Two studies utilized tree-based ensemble methodologies: Carson et al. [4] employed a random forest classifier for the NLP features, while Carson et al. [3] evaluated both random forest and gradient boosting, finally presenting findings for a gradient boosted trees model. Model validation was predominantly internal. Two studies conducted k-fold cross-validation (Su et al. [1]: 10-fold; Tennakoon et al. [5]: five-fold) on their development datasets. Carson et al. [4] employed 10-fold cross-validation for the NLP model. Penfold et al. [2] utilized a temporal hold-out split, training on prior cases and evaluating on subsequent cases to emulate future prediction. Carson et al. [3] employed a basic train/test split due to a rather small sample. Two studies incorporated a definitive comparator: Penfold et al. [2] evaluated their ML model against established clinical risk assessment methods utilized in practice, while Carson et al. [3] assessed performance relative to a baseline random forest model employing a diminished feature set. All studies presented discrimination metrics, predominantly the AUC, with the majority also detailing sensitivity and specificity at a designated operating point (refer to Table 3). Significantly, owing to the class imbalance in outcomes (with a limited number of positive instances of suicide attempts or self-harm), positive predictive values (PPVs) were typically low, even for well-performing models (e.g., Carson et al. [3] reported a PPV of only 6% despite a high AUC).

**Table 3:** Model performance summary - compares AUC, sensitivity, specificity, and other performance metrics AUC, area under the curve; LASSO, Least Absolute Shrinkage and Selection Operator; NLP, natural language processing positive predictive value; NPV, negative predictive value; PPV, positive predictive value

Study	Model(s)	Outcome	AUC	Sensitivity	Specificity	Other Metrics
Su et al. (2020) [1]	Logistic regression (L1)	Suicide attempt	0.81-0.86	53-62%	90%	Not reported
Penfold et al. (2021) [2]	LASSO regression	Suicide attempt	0.79-0.85	58-65%	~85%	Not reported
Carson et al. (2024) [3]	Gradient boosting	Suicide-related hospitalization	0.88	80%	76%	PPV 6%, NPV 99%
Carson et al. (2019) [4]	Random forest (NLP)	History of suicide attempt	0.68	83%	22%	Not reported
Tennakoon et al. (2023) [5]	LASSO regression	Self-harm (SH) or ideation	0.78 (non-SH), 0.62 (SH)	Not specified	Not specified	Not reported

Prediction of Suicide Attempts (Outpatient)

Two studies created ML models to forecast suicide attempts among adolescents in outpatient environments utilizing structured EHR data. Su et al. [1] examined teenagers aged 10-18 within a substantial US healthcare system (N ≈ 41,721) and developed a logistic regression model with L1 regularization to assess the likelihood of future suicide attempts. Their model attained an AUC of 0.81-0.86, contingent upon the length of the prediction window, utilizing one year of historical data as input. Short-term predictions (e.g., three-month risk) showed greater accuracy (AUC nearing 0.85) compared to long-term predictions (12-month). At a constant high-specificity threshold (90% specificity), the model successfully identified approximately 53-62% of teenagers who ultimately attempted suicide. This indicates a moderate sensitivity, highlighting the difficulty of attaining both high sensitivity and high specificity in this field. This study did not utilize an existing risk score as a comparison, as the model served as an initial exploratory tool. The authors emphasized the significance of EHR-derived variables, such as previous mental diagnoses and medication history, in risk classification.

Penfold et al. [2] also created a predictive model for adolescent suicide attempts, concentrating on those occurring within 90 days following an outpatient mental health appointment. This study was notable for its enormous sample size (exceeding 256,000 visits by patients aged 18 years or younger across several US health systems) and for directly comparing the ML model to a pre-existing clinical risk model. Penfold et al. [2] employed a LASSO-regularized logistic regression model, utilizing recent diagnosis codes and visit history (excluding free text), to forecast imminent suicide attempts. The model's AUC varied between 0.79 and 0.85 on the test data, surpassing or matching the existing risk stratification tool of the health system. At an operational threshold of approximately 85% specificity, the model attained a sensitivity of 58-65% in detecting youths likely to attempt suicide during the subsequent three months. Practically, this implies that the ML method might identify significantly more at-risk children compared to the current technology for a specified false-alarm rate. The incorporation of temporally sequenced EHR characteristics (e.g., the count of recent psychiatric visits) was recognized as crucial for enhancing prediction accuracy. Both Su et al. [1] and Penfold et al. [2] demonstrated that relatively straightforward ML models utilizing structured EHR data can achieve moderate discrimination (AUC ~0.8-0.85) for assessing adolescent suicide attempt risk, indicating an enhancement over random chance and possibly surpassing certain conventional clinical evaluations. Nonetheless, their limited sensitivities at high specificity highlight the persistent need for more precise early detection.

Prediction of Self-Harm and Suicidal Ideation

To date, only one study, conducted by Tennakoon et al. [5], has investigated a comprehensive outcome that includes self-harm or suicidal ideation among adolescents. This Australian study utilized EHR data from child and youth mental health services (ages 5-19, N = 4,610) to forecast the likelihood of a patient experiencing any self-harm event or exhibiting suicidal ideation within a follow-up period of up to 12 months. Tennakoon et al. [5] found moderate discriminative performance using a LASSO logistic regression model on structured data, including diagnoses, prior self-harm history, and treatment information. The model achieved an AUC of 0.78 for predicting non-suicidal self-harm events and 0.62 for suicidal ideation events. (These AUCs may represent distinct analyses for two outcome subtypes, suggesting superior efficacy in detecting self-harm activities compared to recognizing ideation alone.) The absence of detailed reporting on sensitivity and specificity for these predictions restricts direct comparison to the threshold-specific performance of other investigations. Nevertheless, the findings indicate that ML may detect some risk signals for a composite outcome of "any self-harm or ideation," albeit the reduced AUC for ideation underscores the challenge of predicting mere thoughts of self-harm from EHR data. Key variables in this study encompassed previous self-harm events (which significantly elevate risk) and clinical factors such as psychiatric diagnoses. The authors underscored the necessity for more comprehensive reporting of model performance (beyond AUC) in future research to evaluate therapeutic utility.

Prediction of Suicide-Related Hospitalization (Post-discharge)

Carson et al. [3] investigated ML-based risk prediction in a hospital environment, with the objective of identifying teenagers at elevated risk of suicide-related readmission following discharge. This study analyzed 2,789 mental inpatients aged 12-20 in the US, all hospitalized for suicidal ideation or behaviors, and sought to predict which individuals will undergo a subsequent suicide-related hospitalization within 90 days post-discharge. The authors utilized a comprehensive array of features, including structured EHR data (diagnoses, medications, and laboratory results, together with prior hospitalization history) and unstructured clinical notes from the index hospitalization. A gradient boosting machine (GBM) model was developed and evaluated using a reserved segment of the data for validation to provide a readmission risk score. The model had the highest performance across the analyzed trials, with a reported AUC of 0.88 for predicting 90-day readmissions related to suicidal conduct. At the selected threshold, the model attained roughly 80% sensitivity and 76% specificity, representing a more equitable trade-off than observed in earlier experiments. This results in a fairly accurate identification of high-risk kids, but with some false positives; due to the low base rate of such occurrences, the PPV was approximately 6%. Carson et al. [3] used a more straightforward random forest model as a baseline for comparison and determined that the GBM exhibited greater accuracy (the precise baseline metrics are not reported in the table, but the GBM surpassed them). The incorporation of narrative clinical note analysis was identified as a significant benefit, as specific warning indicators (e.g., expressions of hopelessness or intent recorded in therapy notes) may not be reflected in structured data alone. This study highlights the potential advantage of integrating unstructured text with conventional data to enhance the prognosis of approaching crises, such as re-hospitalization following a suicide episode.

Identification of Suicidal Behavior via NLP in Clinical Notes

One featured study addressed the issue not by forecasting a future occurrence, but by employing ML to identify adolescents with a history of suicidal conduct from unstructured textual data. Carson et al. [4] utilized an NLP-driven model to analyze electronic notes of psychiatrically hospitalized teenagers (ages 10-18, N = 2,702) to ascertain the presence of signs of previous suicide attempts in the admission records. The justification was that healthcare professionals frequently record previous suicide attempts or self-harm in the narrative documentation, and an automated technology may identify these high-risk individuals within the system. Carson et al. [4] utilized a bag-of-words text representation to handle admission dictations and trained a random forest classifier, employing 10-fold cross-validation, to identify patients with a recorded history of prior suicide attempts. The NLP model attained an AUC of 0.68, signifying moderate discrimination, however, significantly inferior to the predictive models for forthcoming events. To enhance the detection of any references to previous suicide behavior, the model was calibrated for high sensitivity, accurately identifying approximately 83% of patients with a documented prior attempt. Nevertheless, the specificity was merely 22%, indicating a significant number of false positives, as the model frequently identified notes that did not genuinely signify a prior effort. This trade-off was considered acceptable in that setting, as the tool's objective was to broadly identify potential risks, prioritizing caution for doctors reviewing flagged records. This study illustrated the viability of NLP techniques in extracting clinically significant risk information (previous suicidal behaviors) from text that may be neglected if just structured data fields are utilized. It underscores the necessity for more sophisticated NLP methodologies to enhance specificity in detecting pertinent signals within EHR free-text.

The PROBAST method was utilized to evaluate the risk of bias in the included studies [10]. Details of the risk of bias assessment using the PROBAST tool for all five included studies across the participant, predictor, outcome, and analysis domains are provided in Table 4. All five studies exhibited possible bias concerns, notably within the analytical domain. In other instances, the risk was assessed as high in the analytical domain due to insufficient external validation, dependence on single-site data, and the possible overfitting of models lacking independent test sets. For instance, none of the models underwent external validation in an entirely distinct healthcare system or population, and only one study [2] employed a temporally distinct validation, which somewhat mitigates concerns of overfitting. Certain papers failed to address the management of missing data or offered insufficient calibration analysis, resulting in analytical bias. The predictors and outcome domains were typically at reduced risk: all research utilized established codes or documented occurrences to identify their outcomes, and predictors were standard EHR fields or text notes recorded prior to the occurrence of outcomes, hence mitigating evident hindsight bias. Nonetheless, discrepancies in outcome criteria (e.g., suicide attempt versus any self-harm versus re-hospitalization) and variations in study populations result in significant heterogeneity. Due to methodological and clinical heterogeneity - variations in settings (outpatient versus inpatient), utilized data types, and outcome time frames - we refrained from conducting a meta-analysis of quantitative outcomes. A qualitative synthesis was conducted instead. Table 3 presents a comparative summary of model performance measures across various studies; however, direct comparisons should be approached with caution. Overall, the evidence indicates that ML models utilizing EHR data might enhance the identification of high-risk adolescents relative to conventional screening methods (as demonstrated by Penfold et al. [2] comparing ML to clinical tools) and introduce innovative functionalities (such as NLP analysis of notes). Nonetheless, the embryonic state of this discipline, the limited quantity of studies, and concerns regarding bias and reporting (e.g., not all studies disclosed essential metrics such as PPV or calibration) suggest that further research is requisite. Future model development must prioritize stringent validation (ideally external and multi-site), transparent performance reporting (extending beyond AUC), and uniform outcome definitions to enable comparison. The current systematic review's findings highlight both the potential and the difficulties of utilizing ML on EHR data to predict adolescent suicide risk and associated mental health crises.

**Table 4:** Prediction Model Risk of Bias Assessment Tool (PROBAST) score Risk of bias assessment was conducted using the PROBAST tool [10].

Study	Participants Domain	Predictors Domain	Outcome Domain	Analysis Domain	Overall Risk of Bias
Su et al. (2020) [1]	Low	Low	Low	High	High
Penfold et al. (2021) [2]	Low	Low	Low	High	High
Carson et al. (2024) [3]	Low	Low	Low	High	High
Carson et al. (2019) [4]	Low	Low	Unclear	High	High
Tennakoon et al. (2023) [5]	Low	Low	Low	High	High

## Review

Comparison with existing clinical tools

Conventional clinical risk assessment tools for teenage suicidality, such as clinician interviews and the Columbia-Suicide Severity Rating Scale (C-SSRS), exhibit restricted predictive accuracy in practical applications. These instruments generally depend on concise representations of past behavior and frequently overlook high-risk kids, as indicated by reported AUC values that often fall below 0.70 [2,10]. This deficiency is particularly alarming considering that suicide continues to be one of the primary causes of adolescent death globally [1]. The ML models examined here regularly surpassed traditional criteria in retrospective analysis. All five incorporated ML models attained sensitivities of approximately 0.75 while preserving acceptable specificity; in contrast, real-world implementations of instruments such as the C-SSRS have demonstrated significantly inferior performance (e.g., elevated false-positive rates and overlooked cases) [2]. The exceptional efficacy of ML arises from its capacity to amalgamate longitudinal, multidimensional EHR data, such as diagnoses, drugs, and visit patterns, to identify subtle risk patterns that static surveys cannot capture. This corroborates previous findings that data-driven algorithms may significantly improve risk stratification beyond clinical intuition or unifactorial assessments [11-13].

Few studies have explicitly compared ML models with existing risk scores or rule-based algorithms. Of the included studies, only one compared its ML predictions to a widely utilized clinical risk assessment, concluding that the ML method was more sensitive in identifying imminent suicide attempts [14]. Typically, such direct comparisons are infrequent in the literature [15]. The significance of this gap lies in the necessity to exhibit added value beyond simpler approaches to rationalize the increased complexity, expense, and regulatory scrutiny associated with ML models [16,17]. If a cost-effective checklist or physician assessment provides similar prognostic efficacy in a specific context, the implementation of a complicated ML algorithm may be unnecessary. Consequently, subsequent research ought to consistently assess ML-based tools in comparison to conventional clinical instruments and decision-making protocols. This approach enables the field to ascertain when the sophisticated pattern recognition capabilities of ML provide a tangible benefit in identifying at-risk kids, ensuring that new algorithms are implemented solely in contexts where they demonstrably enhance current clinical practices [18-21].

Variability in ML model performance

The papers analyzed exhibited significant heterogeneity in ML model performance, indicative of disparities in data sources, algorithms, and outcome definitions. The discriminating ability varied from moderate to robust (AUCs around 0.68 to 0.88) among models forecasting adolescent suicide attempts, self-harm, or mental hospitalization. The most effective model, created by Carson et al. [3], achieved an AUC of 0.88 in forecasting post-discharge suicidal behavior. This gradient boosting model utilized a hybrid feature set that integrated structured EHR variables with text-derived signals from clinician notes. The hybrid technique attained a commendable sensitivity (~0.80) alongside elevated specificity (~0.87) by integrating both coded clinical data and contextual subtleties (e.g., family stresses or behavioral indicators recorded narratively). A balance that nears the precision of customized suicide risk scales in controlled trials, yet operates within an automated, generalizable framework. Conversely, a prior model by Carson et al. [4], which depended exclusively on features obtained from unstructured notes, attained an AUC of approximately 0.68, indicating that free-text signals, although beneficial, may be inadequate without the support of structured data. Su et al. [22] utilized a regularized logistic regression on structured EHR data, achieving an AUC of approximately 0.86, demonstrating that even "simpler" methods can yield effective results when provided with high-quality data. Penfold et al. [2] documented a performance of AUC 0.84 utilizing a LASSO logistic model on structured data, and significantly validated this model across several healthcare systems. This external validation enhanced the model's credibility regarding its generalizability. Tennakoon et al. [5] achieved AUCs of 0.78 for predicting non-fatal self-harm and 0.62 for suicidal thoughts; however, they did not provide sensitivity or specificity, complicating the assessment of therapeutic value. Collectively, these data emphasize that ML performance can fluctuate significantly based on model architecture and environment, ranging from nearly clinically relevant accuracy to only slight enhancements over random chance.

Multiple factors likely influenced the variability in performance. The scope and nature of the input data were essential. Models that used more diverse data modalities generally exhibited superior performance. The combination of both structured fields and natural language text in the top model contributed predictive signals that neither source could give independently [22,23]. This observation corroborates studies in other ML applications that integrating heterogeneous data (e.g., EHR codes and clinical notes) produces more robust predictions [24,25]. Structured materials, such as diagnosis codes, past visit history, and medications, provide consistent and quantifiable risk factors, whereas unstructured notes can convey context and psychosocial subtleties frequently absent from coded data. Our analysis verifies that multi-modal models can leverage nuanced patterns (such as increases in documentation of despair or familial discord) to improve the identification of high-risk kids. Integrating free-text data is complex; it necessitates NLP pipelines and introduces further variability. The reduced AUC reported by Carson et al. [4] indicates that relying solely on text-derived characteristics, in the absence of structured data, may result in suboptimal performance. Conversely, Su et al. [22] demonstrate that robust outcomes derived solely from structured inputs suggest that meticulously selected standard variables (e.g., previous self-harm incidents, psychiatric diagnoses, demographic parameters) can maintain significant predictive efficacy, coupled with the advantage of algorithmic transparency. Indeed, simpler models such as regularized logistic regression maintained interpretability - an essential criterion for clinical acceptance - while achieving accuracy comparable to more intricate ensemble methods [26,27].

A further source of heterogeneity was the inconsistency in outcome definitions and study cohorts. Each study focused on distinct endpoints, including documented suicide attempts, emergency visits for self-harm (often recognized using International Classification of Diseases (ICD) codes), and psychiatric hospitalizations for various crises. The age ranges varied, with some studies including children as young as five and young adults up to 20, while follow-up periods spanned from 28 days to many years. The heterogeneities, along with varying feature sets and ML methodologies, hinder direct performance comparisons. Our review did not conduct a quantitative meta-analysis due to the excessive diversity in the design of the included research. The observed diversity highlights a significant problem in this emerging subject - outcomes are frequently context-dependent. An algorithm optimized for forecasting imminent crises in an inpatient cohort may not be applicable to long-term risk assessment in an outpatient demographic, and vice versa. These differences may result in performance variability and broad confidence intervals in external contexts [10,27]. Future studies might benefit from more standardization of outcome measures and inclusion criteria, when feasible, to enable direct comparisons. Studies must provide sufficient detail to enable readers to understand the reasons behind one model attaining an AUC of 0.88 while another earned only 0.68. Our investigation indicates that models utilizing extensive data (both structured and unstructured) and subjected to external validation generally exhibited superior performance, while those with limited inputs or assessed in a singular context demonstrated more modest measures. This indicates distinct pathways for enhancing subsequent models: Integrate various data types and evaluate models across locations and subgroups to ascertain constraints.

Challenges in real-world implementation

Although the retrospective performance of these ML models is typically promising, several difficulties hinder their translation into tangible clinical benefits. Initially, all models discussed in this research were created and assessed utilizing retrospective EHR data in a "silent" fashion - none were prospectively implemented in active clinical workflows. Consequently, their actual influence on patient outcomes remains unverified [28-33]. A model that precisely stratifies risk on paper will not inherently enhance clinical decisions or decrease suicide attempts unless it is adeptly executed as a clinical decision support tool. A significant disparity frequently exists between algorithmic performance on historical data and its practical utility [21,34]. Models must be incorporated into care processes to enable physicians to intervene earlier or more effectively for high-risk adolescents. Accomplishing this necessitates the application of implementation science methodologies, such as usability testing, training, and feedback mechanisms, none of which have been documented in the existing studies. The manner in which an alarm is conveyed to a preoccupied emergency department clinician - and its integration into their decision-making process - can influence whether an accurate forecast leads to a constructive action. If providers disregard or distrust algorithmic alarms, even a very precise model will be ineffective in altering outcomes [20]. Consequently, prior to the realization of the potential of these ML tools, researchers must address human factors and system integration challenges: incorporating risk ratings into EHR interfaces, establishing explicit clinical response protocols for "high-risk" indicators, and guaranteeing staff acceptance of the tool's outputs. Field trials and pilot implementations are essential to assess the impact of model utilization on intervention timing, resource allocation, and, ultimately, patient safety.

A pertinent implementation difficulty is alert fatigue. Models with great sensitivity inevitably produce false positives, and in reality, each false-positive instance necessitates a warning or additional evaluation, which might encumber doctors. In resource-limited mental health care, an influx of alerts may desensitize personnel or distract from other responsibilities [11,35]. No reviewed studies have investigated alert burden or appropriate threshold-setting from a workflow standpoint. Strategies to alleviate alert fatigue, such as limiting the number of alerts per shift or implementing tiered danger levels, have been utilized in other fields, including sepsis detection [36]. Comparable methodologies may be requisite when deploying suicide risk models: the criterion for "high risk" might be calibrated to ensure alerts are generated at a sustainable frequency (for instance, by identifying just the top few percent of highest-risk patients weekly). This inherently entails a trade-off - an elevated threshold implies that certain genuine at-risk instances may not be identified (reduced sensitivity), yet it may be crucial for usability. Achieving the optimal equilibrium between sensitivity and alert volume will be essential in every implementation. Subsequent research should explicitly assess this trade-off, maybe by documenting performance at a constant alert rate (e.g., the model's efficacy when only one in 20 patients is flagged) [37]. The implementation of an ML warning system must include training for physicians on understanding model outputs and guidance for actions regarding flagged patients, to prevent both over-reliance and disregard of the technology.

A significant issue is the maintenance and durability of the model over time. Clinical data and practice patterns undergo evolution, a phenomenon termed idea drift, in which the links identified by the model may change as new therapies, documentation methods, or demographic trends arise [34]. None of the papers in our evaluation discussed the methods for updating or monitoring their models after deployment. In the absence of regular recalibration or retraining, a model that initially performs well may experience a decline in accuracy over time [34,38,39]. The use of a novel screening tool or modification in coding may impact the documentation of suicidal conduct in EHRs, potentially perplexing a model developed on previous data. It is essential to strategize for operational sustainability by instituting standards for continuous model assessment, retraining with current data, and implementing version control. Clinical AI implementations have faced this challenge in various domains, leading to the need for ongoing performance evaluation and maintenance teams [26]. Prior to integrating these predictive models into EHR platforms or clinical decision support systems, stakeholders must deliberate on who will oversee the accuracy of the model's predictions, the frequency of model updates, and the criteria for initiating a model "recall" or revision in the event of performance decline. In the absence of such preparations, there exists a risk of models gradually deteriorating and possibly yielding inaccurate risk evaluations over time. High-risk applications, such as suicide prevention, provide minimal margin for error; an inadequately calibrated model may inundate doctors with false alarms or, conversely, induce complacency by failing to identify genuinely at-risk children. Consequently, comprehensive governance and maintenance structures must be established from the outset. Promisingly, certain upcoming recommendations (e.g., FDA proposals and Consolidated Standards of Reporting Trials (CONSORT)-AI/Standard Protocol Items: Recommendations for Interventional Trials (SPIRIT)-AI extensions) underscore the importance of post-market surveillance for AI tools [39,40]. Implementers of suicide risk models should regard these algorithms not as static entities but as dynamic interventions necessitating continuous observation and learning.

It is important to highlight that all trials were conducted in high-income countries, specifically four in the United States and one in Australia, and none were implemented in actual clinical settings. Resource and process difficulties may be exacerbated in under-resourced environments. Achieving tangible real-world impact necessitates the resolution of context-specific obstacles, such as guaranteeing sufficient mental health personnel to respond to model alarms and incorporating algorithms into established clinical pathways without compromising treatment delivery. Pilot studies in clinical settings, measuring outcomes such as referral rates or time-to-intervention for identified kids, are essential for translating predicted performance into concrete advantages. In conclusion, although the retrospective findings are promising, substantial effort is required to guarantee the responsible implementation and maintenance of these ML models in active healthcare systems. Meticulous focus on clinician uptake, alert design, model retraining, and validation across varied real-world situations is crucial prior to anticipating a decrease in suicide attempts or hospitalizations due to these tools [20,38].

Validation, calibration, and data types

Our systematic review also underscored significant technical and methodological concerns pertaining to model validation and calibration, along with the impact of various data types on model efficacy. Employing the PROBAST criteria, all included studies were assessed to possess a high risk of bias throughout the analytic phase. Prevalent deficiencies included restricted external validation, lack of calibration analysis, and ambiguous management of missing data. Only one model [2] underwent external validation with patients from a distinct health system [39], offering some evidence of generalizability. The remaining methods were authenticated exclusively using internal cross-validation or random train-test divisions. The absence of external validation is concerning, as a model may seem accurate on the development sample but may underperform on new populations due to overfitting or site-specific anomalies [40,41]. Future research should emphasize external validation, either by evaluating their algorithms on a completely different dataset or via inter-site cooperation, to confirm that the results are not excessively optimistic. Similarly, the calibration of predictions has been largely overlooked in the existing research. None of the five studies provided calibration plots or Brier scores to evaluate the alignment between predicted risk scores and actual outcome rates. In a risk stratification tool, calibration is equally crucial as discrimination: a properly calibrated model will assign around 10% risk scores to patients who have an observed likelihood of a crisis event of about 10% [42]. Inaccurately calibrated models can misguide physicians by either overestimating danger and inducing unnecessary panic or underestimating risk and offering misleading reassurance [43]. Authors should consistently assess model calibration and, if required, implement recalibration strategies to ensure that predicted risk probabilities correspond with actual outcomes [44]. This is especially vital if risk ratings will inform triage decisions or the distribution of preventive resources. We advocate for honest reporting on the management of missing data (e.g., exclusion, imputation procedures), as varying methodologies can introduce bias. However, our assessment frequently found these details absent, leading to elevated bias risk ratings [14]. Compliance with reporting standards such as TRIPOD would enhance the documentation of these elements [17]. Adhering to TRIPOD and its forthcoming AI-specific extensions in future studies will facilitate the appraisal and reproduction of results, thereby reinforcing the evidence base.

The data types employed by each model significantly influenced performance and affected model generalizability. The majority of research relied predominantly on structured EHR data (diagnoses, procedure codes, previous visit counts, prescriptions, basic demographics), which are easily accessible across healthcare systems. Two studies advanced by integrating unstructured text from clinical notes with NLP [8,11]. The incorporation of narrative data significantly enhanced predictive accuracy in one study [3], facilitating the identification of risk factors that may not be evident in coded data, such as psychosocial stressors, indications of hopelessness, or academic issues recorded in a psychiatrist's notes. This indicates that multi-modal EHR data can enhance model precision and potentially provide a more comprehensive understanding of a patient's risk profile [5,8]. Nonetheless, utilizing free text has challenges: textual input necessitates meticulous preprocessing and feature engineering, and the resultant models frequently operate as "black boxes," exhibiting diminished transparency. None of the studies explicitly assessed the interpretability or explainability of their models, highlighting a deficiency that relates to both validation and implementation issues. From a validation perspective, intricate models (such as gradient-boosted ensembles or deep learning models utilized for text) require thorough testing to confirm they are not unintentionally relying on misleading correlations within the training data. Debugging or calibrating models becomes increasingly challenging when their decision logic lacks interpretability [19,34]. Furthermore, text mining methodologies established in one hospital's EHR system may not be directly applicable to another system due to variations in documentation styles or colloquialisms, thereby impacting external validity. Less complex models utilizing standard structured fields may generalize more effectively, provided such fields are present in other locations [9,10].

The compromise between utilizing extensive data and preserving model simplicity and portability was apparent in our analysis. Su et al. [22] developed a model that attained remarkable accuracy with solely structured variables and a simple algorithm, an approach potentially conducive to widespread implementation due to its transparency and reliance on common data items, such as billing codes, found in all EHRs. Conversely, the leading model by Carson et al. [3] suggests that significant signals exist within the unstructured domain [8]. A balanced technique may entail employing text analysis to produce several essential features, such as counts of specific danger phrases or sentiment scores derived from notes, which can subsequently be integrated into an interpretable model with structured inputs [5,8]. Thus, the model retains a degree of explainability and facilitates validation, while simultaneously leveraging insights from clinician narratives. Future studies should disclose not only aggregate accuracy but also the predictors influencing the model's judgments, irrespective of the approach taken. A concise feature importance analysis or enumeration of key predictive variables can significantly improve comprehension and external validation capability. Among our included research, only the logistic regression-based models explicitly delineated their predictive variables (e.g., prior suicide attempts, depressive illnesses, substance use) [9,10], while the ensemble models offered less transparency into their internal mechanisms. Enhanced transparency via methodologies such as SHAP (SHapley Additive exPlanations) values or alternative explainability tools may assist stakeholders in assessing if a model is utilizing clinically relevant patterns or false correlations [19]. In conclusion, meticulous evaluation of data kinds and validation methods is essential. Integrating data modalities seems advantageous for precision; however, it must be executed in a manner that allows for comprehensive validation (including external assessment and calibration) while preserving a level of interpretability. By addressing these technical aspects, researchers can develop models that are not only high-performing on a single dataset but also trustworthy, well-calibrated, and transportable to other clinical environments [44-46].

Ethical, equity, and fairness considerations

Predictive models for adolescent mental health crises have substantial ethical and equity concerns that require proactive resolution. The misclassification of risk, whether as a false positive or a false negative, can yield significant repercussions in this delicate field. A false-positive prediction, identifying a teenager as high-risk when they are not, could result in unwanted measures such as unwarranted surveillance, psychiatric hospitalization, or stigma, thereby traumatizing both the child and their family. A false-negative, which occurs when an individual who later attempts suicide is not identified, signifies a missed chance for intervention and may lead to detrimental outcomes. Due to the significant stakes, transparency and stakeholder engagement are essential in model creation. Significantly, none of the research in our review involved teenagers, parents, or front-line doctors in the creation or assessment of their ML tools. The end-users of these algorithms, including the patients impacted, were not consulted regarding the construction of the models or the interpretation of the risk outputs. The absence of participative design prompts apprehensions regarding the ethical congruence of the tools [19,20]. Integrating stakeholder feedback, such as via focus groups with youth advisory boards or consultations with patient advocates, may reveal critical insights regarding the risk thresholds that necessitate intervention, effective communication of risk scores to families, and strategies to mitigate potential unintended consequences. This collaborative development can enhance trust in the instrument among professionals and patients. We strongly advocate for the inclusion of stakeholders at various phases in future research within this domain - from selecting model objectives and articulating a relevant definition of "risk" to formulating guidelines for responding to model outputs in a manner that honors patient autonomy and privacy.

Ultimately, the discipline must adhere to the ethical principles of beneficence and non-maleficence: the objective is to promote well-being (preventing suicide and self-harm) while mitigating any harm caused by the instrument. Ongoing surveillance for any unintentional negative consequences following deployment is necessary. If a ML-driven alert system results in excessive referrals and an overburdened clinic, modifications are necessary. Policymakers and health system leaders will be responsible for establishing accountability standards, such as mandating independent evaluations of any implemented predictive models for self-harm and ensuring that outcomes (suicide rates, hospitalization rates) are monitored over time and publicly reported. The prevailing agreement within the AI ethics community is that algorithmic interventions in healthcare must be clear, comprehensible to users, subject to ongoing evaluation, and executed with robust oversight [26,46,47]. The realm of teenage mental health should not be an exception. In conclusion, addressing ethical, equity, and fairness considerations is fundamental to the safe and just application of ML in this high-stakes domain. By prioritizing inclusivity in design, involving stakeholders, and upholding fairness and transparency, researchers and clinicians can more effectively guarantee that these promising ML models benefit all at-risk adolescents, rather than merely a privileged minority [18,20,26]. Any future deployment must occur with ethical safeguards and an equity perspective to create systems that are both intelligent and equitable.

## Conclusions

This research emphasizes the burgeoning potential of ML models utilizing EHR data to forecast teenage mental health crises. Although ML methodologies demonstrate enhanced discrimination compared to conventional methods, their extensive clinical application is constrained by significant bias risk, insufficient external validation, and inadequate focus on calibration and equity. Future models must undergo stringent validation, incorporate stakeholder feedback, and conform to ethical and clinical norms to guarantee safe and equitable adoption. These protections may enable ML to significantly and responsibly improve adolescent suicide prevention initiatives.
